# Contouring lumbosacral plexus nerves with MR neurography and MR/CT deformable registration technique

**DOI:** 10.3389/fonc.2022.818953

**Published:** 2022-11-09

**Authors:** Xi Cao, Xian-Shu Gao, Wei Li, Peilin Liu, Shang-Bin Qin, Yan-Bin Dou, Hong-Zhen Li, Shiyu Shang, Xiao-Bin Gu, Ming-Wei Ma, Xin Qi, Mu Xie, Dian Wang

**Affiliations:** ^1^ Department of Radiation Oncology, Peking University First Hospital, Beijing, China; ^2^ Department of Radiology, Peking University First Hospital, Beijing, China; ^3^ Department of Oncology, Hebei North University, Zhangjiakou, Hebei, China; ^4^ Department of Radiation Oncology, First Affiliated Hospital of Zhengzhou University, Zhengzhou, Henan, China; ^5^ Department of Radiation Oncology, Rush University Medical Center, Chicago, IL, United States

**Keywords:** lumbosacral plexus, contouring, radiation-induced plexopathy, MR neurography, multi-modality registration

## Abstract

**Purpose:**

It is difficult to contour nerve structures with the naked eye due to poor differentiation between the nerve structures with other soft tissues on CT images. Magnetic resonance neurography (MRN) has the advantage in nerve visualization. The purpose of this study is to identify one MRN sequence to better assist the delineation of the lumbosacral plexus (LSP) nerves to assess the radiation dose to the LSP using the magnetic resonance (MR)/CT deformable coregistration technique.

**Methods:**

A total of 18 cases of patients with prostate cancer and one volunteer with radiation-induced lumbosacral plexopathy (RILSP) were enrolled. The data of simulation CT images and original treatment plans were collected. Two MRN sequences (Lr_NerveVIEW sequence and Cs_NerveVIEW sequence) were optimized from a published MRN sequence (3D NerveVIEW sequence). The nerve visualization ability of the Lr_NerveVIEW sequence and the Cs_NerveVIEW sequence was evaluated *via* a four-point nerve visualization score (NVS) scale in the first 10 patients enrolled to determine the better MRN sequence for assisting nerve contouring. Deformable registration was applied to the selected MRN sequence and simulation CT images to get fused MR/CT images, on which the LSP was delineated. The contouring of the LSP did not alter treatment planning. The dosimetric data of the LSP nerve were collected from the dose–volume histogram in the original treatment plans. The data of the maximal dose (D_max_) and the location of the maximal radiation point received by the LSP structures were collected.

**Results:**

The Cs_NerveVIEW sequence gained lower NVS scores than the Lr_NerveVIEW sequence (Z=-2.887, p=0.004). The LSP structures were successfully created in 18 patients and one volunteer with MRN (Lr_NerveVIEW)/CT deformable registration techniques, and the LSP structures conformed with the anatomic distribution. In the patient cohort, the percentage of the LSP receiving doses exceeding 50, 55, and 60 Gy was 68% (12/18), 33% (6/18), and 17% (3/18), respectively. For the volunteer with RILSP, the maximum irradiation dose to his LSP nerves was 69 Gy.

**Conclusion:**

The Lr_NerveVIEW MRN sequence performed better than the Cs_NerveVIEW sequence in nerve visualization. The dose in the LSP needs to be measured to understand the potential impact on treatment-induced neuropathy.

## Introduction

The use of stereotactic body radiation therapy (SBRT) has been increasing for the treatment of abdominal and pelvic malignancies, including prostate cancer and its abdominal/pelvic lymphatic drainage sites (1–4). Although SBRT showed comparable outcomes and toxicities with intensity-modulated radiation therapy in general, there are reports that some patients treated with SBRT developed neurological symptoms, which has prompted us to better delineate the lumbosacral plexus (LSP) for accurate dosimetry analysis during the high-dose irradiation of pelvic tumors (5).

Radiation-induced lumbosacral plexopathy (RILSP) is rare but can cause severe signs and symptoms following pelvic radiation, which often manifests as lower leg pain, numbness, weakness, and paralysis in extreme cases (6). It has been assumed that the pathogenesis of RILSP is related to the fibrosis of the neurovascular bundle following radiation therapy (RT) (7). Limiting the dose to the LSP is therefore considered to reduce the incidence of RILSP, which requires an accurate delineation of the LSP for dosimetric analysis during the high-dose irradiation of pelvic tumors.

The challenge of LSP contouring lies in the poor differentiation between nerves and other soft tissues with similar Hounsfield unit (HU) values (e.g., vessels and muscles) on simulation CT (8). There are generally two methods to contour the LSP at present. The first method is to contour a range of LSPs using anatomical reference points on CT (7). The second method is to contour single or multiple nerves under the guidance of MR *via* image fusion techniques. The former method requires the knowledge of gross/imaging anatomy, which is often not precise, whereas in the latter method, finding the nerve structures on traditional MRI sequences (without the specialized nerve visualization method) is also not easy.

MR neurography (MRN) is a specialized MR technique that visualizes the peripheral nerves *via* suppressing the signals of the fat and blood flow, with the advantage of a high contrast of nerve signals relative to other soft tissues, enabling physicians to identify and track nerve travel more easily on MR (9–11). Furthermore, considering the fact of the change in the spinal curvature between CT and MR examinations, rigid registration may not achieve satisfying results. Deformable registration can match organs with position or shape variations due to body position changes and has been widely applied during clinical RT practice (12–14). The combination of the two novel techniques may be able to address the challenges of LSP nerve contouring. The purpose of this study is to identify one special MR neuroimaging sequence to better delineate the LSP to assess the radiation dose to the LSP using an MR/CT deformable coregistration technique.

## Materials and methods

### Participants

**Figure 1:**
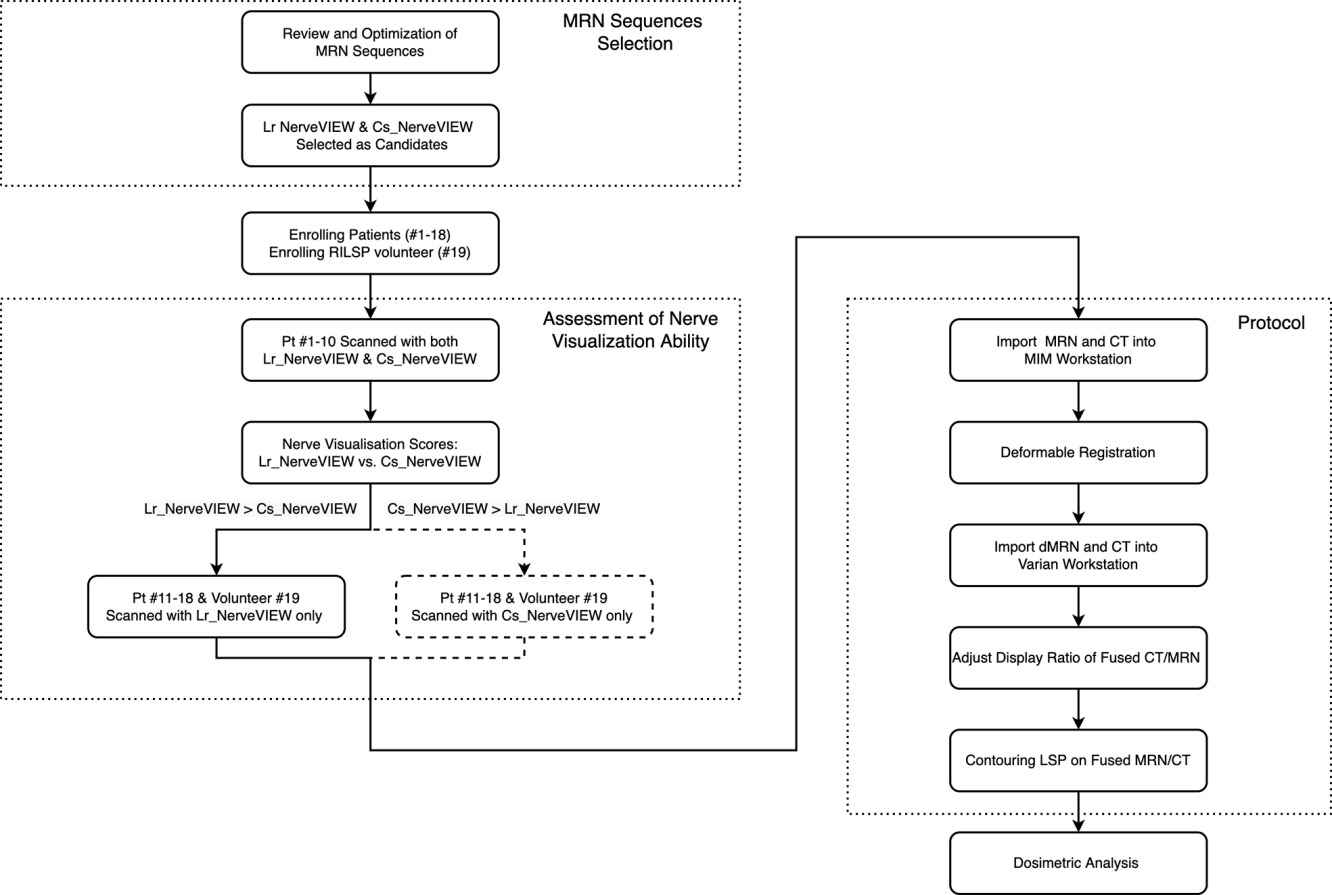
Study design flowchart. The flowchart boxes represented by dashed lines refer to the case when cs_NerveVIEW performed better than Lr_NerveVIEW in the assessment of nerve visualization ability, which did not occur in this study. Pt #., patient number; dMR, deformed MR.

This prospective study was approved by the Institutional Review Board of the Peking University First Hospital (No. 2021-313). The study design is shown in **Figure 1**.

Any adult patients with prostate cancer who were required to receive radiation treatment to the pelvis and gave consent to MRN examination were eligible for this study. The inclusion criteria included simulation CT and MRN images ranging from minimally T12 vertebrae to the femoral neck and treatment plan with dose constraints to the conventional pelvic organ at risk (OAR) only (not including dose constraints to LSP nerves). Additional exclusion criteria were general contraindications to MR imaging (e.g., claustrophobia and metal implants) and an interval between MR and CT scanning of more than 2 weeks.

In addition to the patient cohort, one volunteer with RILSP also participated in this study. This volunteer was diagnosed with prostate cancer with bone metastases to the right sacrum and the left iliac bone and treated with RT to both the primary site and the metastatic lesions. One month after the completion of RT, the patient developed mild persistent motor weakness in both lower extremities, which is considered as RILSP.

The MRN sequence was applied to 18 patients (patient #1 to #18) as well as the volunteer (#19) with RILSP. To determine the better MRN sequence for this study, the first 10 patients enrolled (#1 to #10) underwent MRN examinations with two different MRN sequences (Lr_NerveVIEW sequence and Cs_NerveVIEW sequence). After determining the sequence that was performing better in the nerve visualization ability, the remaining eight patients (#11 to #18) and the volunteer (#19) underwent this one particular sequence only. Tumor, imaging, and treatment details for all 19 participants are summarized in **Table 1**.

**Table 1:** Participants, tumor, imaging, and treatment.

No.	MRN NerveVIEW	TNM Stage	Purpose	GTV	Dose	Interval
1	Lr and Cs	cT4N0M0	Radical	Prostate and seminal sac	70Gy/25f; 2.8 Gy/f	3d
2	Lr and Cs	cT2N0M0	Radical	Prostate and seminal sac^∫^	70Gy/25f; 2.8 Gy/f	0d
3	Lr and Cs	cT2NxM0	Radical	Prostate and seminal sac	70Gy/25f; 2.8 Gy/f	1d
4	Lr and Cs	cT2cN0M0	Radical	Prostate and seminal sac^∫^	70Gy/25f; 2.8 Gy/f	1d
5	Lr and Cs	pT3bN0M0	Adjuvant	Tumor bed and seminal sac	62.75Gy/25f; 2.5 Gy/f	1d
6	Lr and Cs	cT3bN0M0	Radical	Prostate and seminal sac	70Gy/25f; 2.8 Gy/f	10d
7	Lr and Cs	pT2cN0M0	Adjuvant	Tumor bed and seminal sac	62.75Gy/25f; 2.5 Gy/f	14d
8	Lr and Cs	cT3bN0M0	Radical	Prostate and seminal sac	70Gy/25f; 2.8 Gy/f	1d
9	Lr and Cs	cT2cN0M0	Radical	Prostate and seminal sac	70Gy/25f; 2.8 Gy/f	2d
10	Lr and Cs	cT2aN0M0	Radical	Prostate and seminal sac^∫^	70Gy/25f; 2.8 Gy/f	11d
11	Lr	pT2cN0M0	Adjuvant	Tumor bed and seminal sac	62.75Gy/25f; 2.5 Gy/f	1d
12	Lr	cT2aN0M0	Radical	Prostate^∫^	67.5Gy/25f; 2.7 Gy/f	2d
13	Lr	cT1bN0M0	Radical	Prostate and seminal sac	67.5/25f; 2.7 Gy/f	0d
14	Lr	cT4N1M0	Radical	Prostate, seminal sac and iliac mLNs	70Gy/25f; 2.8 Gy/f	1d
15	Lr	cT2cN0M0	Radical	Prostate and seminal sac^∫^	70Gy/25f; 2.8 Gy/f	5d
16	Lr	cT4N1M0	Radical	Prostate, seminal sac and pelvic mLNs	70Gy/25f; 2.8 Gy/f	2d
17	Lr	cT4N1M0	Radical	Prostate, seminal sac and pelvic mLNs	70Gy/25f; 2.8 Gy/f	0d
18	Lr	cT3bN1M1	Radical	Prostate, seminal sac and pelvic mLNs Left iliac bone metastasis	70Gy/25f; 2.8 Gy/f 50Gy/10f; 5 Gy/f	4d
19	Lr	cT2cN0M1	Radical	Prostate and seminal sec Bone metastases	70Gy/25f; 2.8 Gy/f 65Gy/25f; 2.6 Gy/f	/

No., patient number; GTV, gross tumor volume; CTV, clinical target volume; mLNs, metastatic lymph nodes; Interval, the time interval between the date of simulation CT and MRN. A total of 15 patients in this cohort received radical RT, and 3 patients received adjuvant RT after prostatectomy. The median time interval of CT and MR scanning was 1.5 days.

^∫^ No prophylactic whole-pelvic radiation. Otherwise, treated with whole-pelvic radiation (47.5Gy/15f) simultaneously.

### Imaging technique and magnetic resonance neurography sequences

All MRN images were obtained from a Philips Ingenia 3.0 T (Philips Healthcare, Amsterdam, Netherlands) using a dStream Torso phased array coil. The patients were scanned in the supine position on a plane table with arms crossed on the chest, ranging from the T12 vertebrae to the superior border of the femoral neck. The plane table was used to mitigate the spinal curvature change between CT simulation and MRN examinations.

Two MRN sequences (Lr_NerveVIEW sequence and Cs_NerveVIEW sequence) were established from the optimization of the parameters of an existing MRN sequence, the 3D NerveVIEW sequence (9). The Lr_NerveVIEW sequence is a heavy T2-weighted fast spin-echo MRN sequence with a small voxel of 1.22 mm × 1.25 mm × 2.00 mm. The short tau inversion recovery technique was used as a fat suppression method, and the motion-sensitized driven-equilibrium technique was used to suppress the signals of blood flow to further enhance the contrast of nerve signals to the background. Compared with the 3D NerveVIEW sequence, the field of view of Lr_NerveVIEW was extended to cover from the T12 vertebral body superiorly, the superior margin of the femoral neck inferiorly, the anterior superior iliac spine anteriorly, and the entire lumbosacral foramens posteriorly. The voxel of the Cs_NerveVIEW sequence was further narrowed to 1.2 mm isotropically on the base of the Lr_NerveVIEW sequence to investigate whether a smaller voxel could result in better nerve visualization. To compensate for the weakening of the signal intensity and the prolonged scanning time due to voxel reduction (15, 16), the spectral attenuated in-version recovery technique and the compressed sensing technique were applied to improve the signal-to-noise ratio and reduce the scanning time, respectively.

### Evaluation of nerve visualization ability

The MRN images of patients #1 to #10 were reviewed on a PACS system (Philips Healthcare, Netherlands) by a radiation oncologist with over 10 years working experience (XBQ) and a resident (XC). The ability of nerve visualization was evaluated using a four-point grading scale [nerve visualization score (NVS)]: 4, excellent (the entire LSP structure is clearly visualized and is of excellent signal intensity); 3, good (the entire LSP structure is visualized and is of good signal intensity); 2, moderate (a part of the LSP structure is visualized and is of moderate signal intensity); and 1, poor (the LSP structure is not visualized and is of poor signal intensity) (17).

### Delineation of lumbosacral plexus with magnetic resonance/CT deformable registration

The MRN sequences with better performance in terms of nerve visualization and simulation CT were imported into the software package MIM (V6.9.4, MIM Software Inc., Cleveland, OH, USA). The workflow “MR to CT—Deformable Registration (Multi-modality method)” was used to achieve the deformable registration of two sets of images. The aligned secondary image (deformed MR), as well as the original simulation CT, was sent to Eclipse v13.5 (Varian Medical Systems, CA, USA).

**Figure 2:**
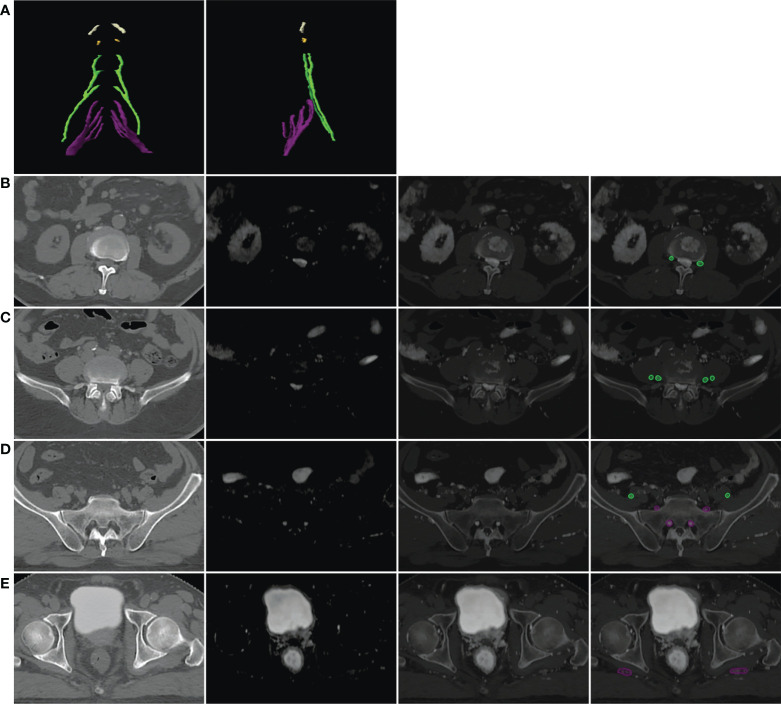
Example of the lumbosacral plexus (LSP) in three-dimensional (3D) and axial views. **(A)** LSP in the 3D view: white, T12 nerve root; yellow, L1 nerve root; green, L2–L4 nerve roots and femoral nerve; purple, L5–S3 nerve roots and sciatic nerve. **(B–E)** Axial view of the LSP on the level of L2 intervertebral foramina **(B)**, L4 intervertebral foramina **(C)**, sacrum **(D)**, and the femoral head **(E)**. The first column presented the original simulation CT images. The second column presented deformed MR in the axial view. The third column presented the automatically registered CT and deformed MR images on Eclipse with the display ratio adjusted to a state in which the nerves “light up” on the fusion images. The last column presented the contours of nerves on fusion images.

Deformed MR and simulation CT were automatically registered in Eclipse because they shared the same DICOM frame of reference. The windows and the display ratio of the two images were adjusted to a state in which the nerves “light up” on the CT images (**Figure 2**). The nerves were contoured by the following steps. First, contour the L2–L4 nerve roots on the axial view and track down (move the plane downward) to contour the femoral nerve as far as possible (the structure contoured in this step was named as “FN”). Second, contour the L5 to S3 roots and track down to contour the sciatic nerve to the superior border of the femoral neck (the structure contoured in this step was named as “SN”). Sometimes, the coronal view may be used to determine the confluence points of the nerve roots and trace these nerves’ travel if unclear on the axial view. Finally, move the plane to the top and contour the L1 and T12 roots.

### Dosimetric data from dose–volume histogram

All dosimetric analyses were done retrospectively without influencing the original treatment dose planning. The prescription isodoses of the original treatment plan were transferred from Eclipse v13.5. The volumes of the LSP, FN, and SN were calculated. The maximum dose, the mean dose of the LSP, and the percentage of the volume receiving dose ≥50, ≥55, and ≥60 Gy were calculated and recorded. The equivalent dose in 2 Gy per fraction (EQD2) was calculated using a linear quadratic model with α/β_peripheral nerve_ = 2 (18).

### Statistical analysis

All analyses were done using SPSS v24.0 (IBM, Armonk, NY, USA). A nonparametric test for two related samples (Wilcoxon signed mean rank test) was performed with the NVS during the comparison of the nerve visualization ability, with p < 0.05 considered statistically significant. Descriptive statistics were performed with the LSP volume and DVH parameters for patients #1–18 and the volunteer #19.

## Results

The whole LSP structures were successfully created in 18 patients and one volunteer utilizing the method described above. An example of this OAR structure in the axial view and the three-dimensional (3D) view is shown from patient #1 in **Figure 2**.

### Nerve visualization

**Figure 3:**
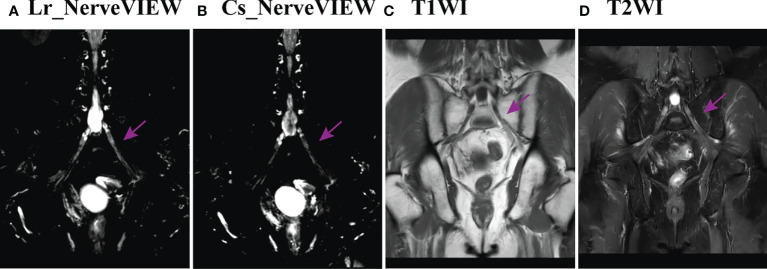
Example of the LSP structure in Lr_NerveVIEW **(A)**, cs_NerveVIEW **(B)**, T1-weighted **(C)** sequences, and T2-weighted fat-suppressing sequence **(D)** in the coronal view from patient #7. The purple arrow indicated the left S1 nerve root. The S1 nerve root was clearly seen on Lr_NerveVIEW and Cs_NerveVIEW sequences but appeared slightly “brighter” on the Lr_NerveVIEW sequence. Meanwhile, the same nerve root on T1WI and T2WI sequences appeared vaguer and had worse differentiation with surrounding tissues.

Of the 20 MRN images of patients #1–10, 19 were scored as “4” or “3” by two observers, which means that 95% (19/20) of the MRN images showed clear LSP nerve structures with an excellent or good intensity of signals. There was no statistical difference in the NVS between the two observers (Z = -1.633, p > 0.05). However, Lr_NerveVIEW gained a higher score than cs_NerveVIEW (median: 4, average: 3.65 *vs*. median: 3, average: 3.15, Z = -2.887, p = 0.004), which indicated that the Lr_NerveVIEW sequence performed better in this study. An example of MRN and traditional MR sequences is shown from patient #7 in **Figure 3**.

### Dose–volume histogram parameters

**Figure 4:**
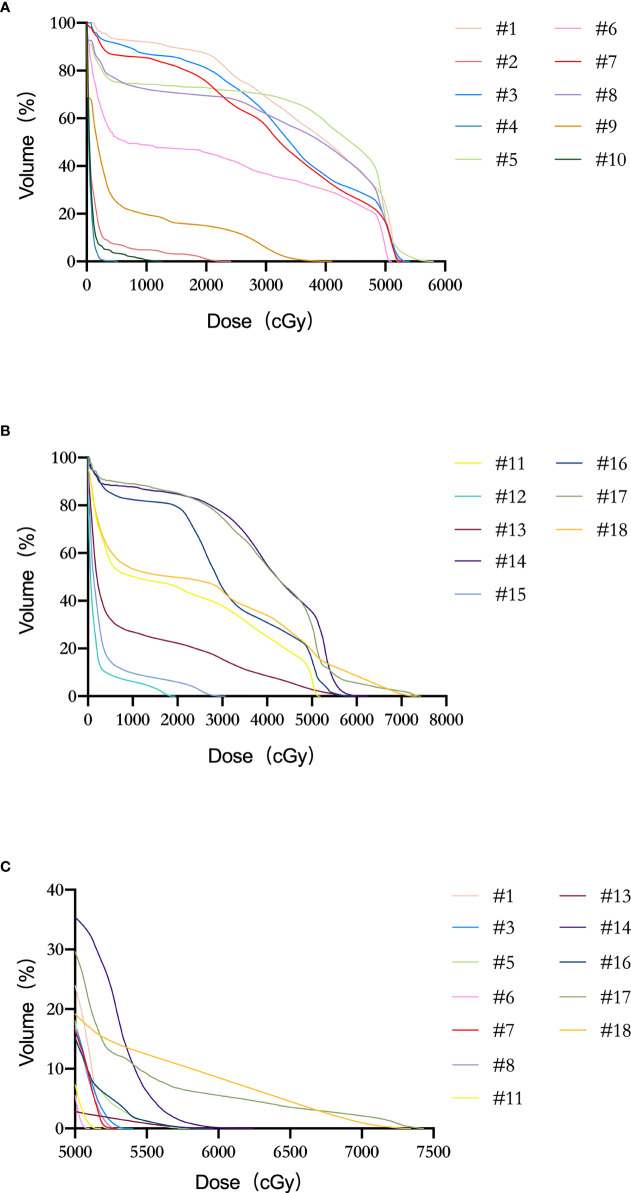
Dose–volume histogram (DVH) of LSP nerves for patients #1–18. **(A)** Patients #1–10. **(B)** Patients #11–18. **(C)** Patient with the dose delivered to LSP ≥ 50 Gy.

**Figure 5:**
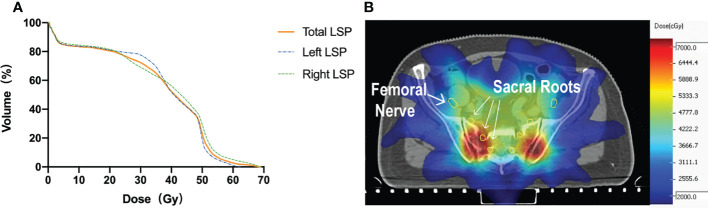
**(A)** DVH of the volunteer (#19) with radiation-induced nerve injury after radiation. **(B)** Isodose level lines and contours of the nerves on simulation CT. Yellow circle: the femoral nerves and sacral nerve roots.

In the patient cohort (#1–18), the mean LSP volume, the mean FN volume, and the mean SN volume were 52.6 ± 6.5 cm^3^ (range, 41.4–68.2 cm^3^), 17.5 ± 3.2 cm^3^ (range, 13.2–23.5 cm^3^), and 33.4 ± 3.7 cm^3^ (range, 27.0–42.0 cm^3^), respectively. The median maximal dose to the LSP was 53.0 Gy (range: 5.1–74.3 Gy). The percentage of patients whose LSP nerves received a dose over 50, 55, and 60 Gy was 68% (12/18), 33% (6/18), and 17% (3/18), respectively. After excluding the five patients who did not receive prophylactic whole-pelvic radiotherapy (WPRT), the percentage of patients receiving doses exceeding 50, 55, and 60 Gy to the LSP nerves was 92% (12/13), 46% (6/13), and 23% (3/13), respectively (**Figure 4**). For the volunteer (#19), the maximum doses occurred at the right (69 Gy in actual dose, EQD2 79.4 Gy) and left (62 Gy in actual dose, EQD2 71.3 Gy) sacral nerve roots, respectively (**Figure 5**).

## Discussion

It has been long recognized that high-dose radiation therapy can cause neurotoxicity (19). To avoid delivering unexpected high-dose radiation to the LSP nerves, accurate contouring is the first and foremost condition. In this study, we identified the Lr_NerveVIEW sequence as a suitable MRN sequence for LSP nerve contouring and successfully contoured the LSP nerve structures in 19 participants based on the MRN/CT deformation registration technique. Further dosimetric analysis showed that a considerable proportion of patients (3/13) who underwent regular pelvic radiation had unexpected high-dose radiation to their LSP nerves, which could have been avoided if the LSP nerves were contoured and protected during treatment planning. This is also the case for the patient with RILSP.

In 2012, Sun K. Yi et al. first proposed an empirical method for contouring the LSP based on anatomy and imaging atlas (20). Anatomic structures such as the great vessels and muscles were used as reference points to define the range of LSPs. Although it is a pioneering proposal, there are still several problems associated with complex pelvic anatomy such as a steep learning curve and poor contouring consistency in certain areas (21). Our protocol integrates the advantage of the intuitive nerve visualization of MRN into simulation CT directly, which lowers the requirement for the knowledge of the complex anatomy. It is also not necessary to contour the whole LSP in a case when the tumor is only close to a certain nerve root or nerve. Our protocol enables physicians to personalize the contouring of the LSP as indicated.

Studies concerning radiation-induced plexopathy have been mainly focused on the brachial plexus (BP) in recent years. While contouring guidelines and dose constraints for BP have been well developed (22–24), there are few studies focused on the LSP. Due to the lack of a widely acknowledged method for LSP nerve contouring in the past, the relationship between the dose to LSP nerves and the development of LSP nerve injury was poorly understood. The incidence of RILSP is approximately 1.3%–6.7% (25, 26), but this rate might be underestimated due to the lack of routine screening (6). Most RILSP cases occur when D_max_ exceeds 60 Gy (6, 27), but nerve injury can also occur at a maximum dose at as low as 37 Gy (28). Taking the tolerance dose for BP, 66 Gy in conventionally fractionated RT, as a reference (22, 23), it may be reasonable to constrain the LSP dose to 60–66 Gy.

To the best of our knowledge, this is the first study that used MRN to aid the contouring of LSP nerves. Traditional MRI sequences were mainly used in previous studies about MRI-assisted nerve contouring (29–32). A 3D MRN sequence such as Lr_NerveVIEW with superior nerve-to-background contrast could provide improved assistance in LSP nerve contouring. Furthermore, the scanning time of the Lr_NerveVIEW sequence was 7 min and 40 s, which is more clinically acceptable compared with the sequences of research purpose with even smaller voxels but longer scanning time (33). Another originality of this study lies in the deformable registration technique to achieve the precise fusion of images (**Supplement Table 2**). The use of deformable registration addresses the limitation of rigid registration in real clinical scenarios; that is, the patient is rarely in the same position during two examinations because thermoplastic masks are not used during MR scanning. Compared with rigid registration, deformable registration can provide a better localization of LSP nerves.

One limitation of our study is the lack of direct comparison of MRN and traditional MRI sequences. Although not supported by data, MRN does outperform traditional MRI sequences in nerve visualization based on clinical experience and a direct impression of MR images. Additionally, although MRN sequences are available in routine clinical protocols, they are not as widely used as traditional MR sequences. There is also a concern about the economic burden of this additional examination. Both factors limit the wide application of this MRN-based protocol to the daily clinical routine. However, for those who prioritize the protection of LSP nerves (e.g., patients with long life expectancy or patients with strong desire for nerve function preservation), they should be fully informed of this option to perform an MRN as an aiding method to contour LSP nerves for protection.

It is also unclear whether a large dose of radiation delivered to these smaller nerve branches can cause severe neurologic symptoms or not. Even with the Lr_NerveVIEW sequence, we are still unable to assess smaller nerve branches due to the inadequate spatial resolution and suppression of slow-flowing vessels. To address this challenge, more advanced imaging techniques, such as the use of gadolinium-based contrast agents to improve small nerve conspicuity, could be implemented (34).

The small number of patients enrolled is also one of the limitations. Moreover, the cases enrolled all received moderately hypofractionated radiation therapy, although the contouring of the LSP may be more meaningful in the setting of SBRT because SBRT provides an even higher biological equivalent dose. However, considering that this study focused only on the contouring, whether patients received MHRT or SBRT did not affect the conclusion. As for treatment planning, we are now working on collecting more SBRT cases and hope that this method still shows its superiority in correlation with clinical outcomes.

## Conclusions

This study identified the Lr_NerveVIEW sequence as a suitable MRN sequence to aid the contouring of LSP nerves. Contouring LSP nerves *via* the MRN/CT deformable registration technique is practical and operable.

## Data availability statement

The raw data supporting the conclusions of this article will be made available by the authors, without undue reservation.

## Ethics statement

The studies involving human participants were reviewed and approved by Peking University First Hospital. The patients/participants provided their written informed consent to participate in this study.

## Author contributions

Study conception and design: X-SG, DW, and XC. Data acquisition: WL, S-BQ, Y-BD, H-ZL, SS, M-WM, XQ, and MX. Data and statistical analysis: XC and PL. Drafting of the manuscript: XC and S-BQ. Critical editorial and writing contributions: X-SG, DW, and XC. All authors contributed to the article and approved the submitted version.

## Funding

We are grateful to the funding provided by China International Medical Foundation (grant number: 2019-N-11-08).

## Acknowledgments

The authors greatly thank the Department of Radiology, Peking University First Hospital, for teaching MRN interpretation and reserving MR time slots for patients participating in this study.

## Conflict of interest

The authors declare that the research was conducted in the absence of any commercial or financial relationships that could be construed as a potential conflict of interest.

## Publisher’s note

All claims expressed in this article are solely those of the authors and do not necessarily represent those of their affiliated organizations, or those of the publisher, the editors and the reviewers. Any product that may be evaluated in this article, or claim that may be made by its manufacturer, is not guaranteed or endorsed by the publisher.

## Abbreviations

D_max_, maximal dose; DVH, dose–volume histogram; EQD2, the equivalent dose in 2 Gy per fraction; FN, L2–L4 nerve roots plus femoral nerve; LSP, lumbosacral plexus; MRN, magnetic resonance neurography; NVS, nerve visualization score; OAR, organ at risk; RILSP, radiation-induced lumbosacral plexopathy; SBRT, stereotactic body radiation therapy; SN, L5-S3 nerve roots plus sciatic nerve.
