# Supplementary material for: Contouring lumbosacral plexus nerves with MR neurography and MR/CT deformable registration technique
Source: Front Oncol. 2022 Nov 9;12:818953. doi: 10.3389/fonc.2022.818953 (PMC9683691; doi:10.3389/fonc.2022.818953)
Supplement: Supplementary file 1 [file Table_1.docx]

Supplementary Material

**Table A1. Parameters of Lr_NerveVIEW and the Cs_NerveVIEW**

|  | Lr_NerveVIEW | Cs_NerveVIEW |
| --- | --- | --- |
| Orientation | Coronal | Coronal |
| Acquired (mm) | 1.22 x 1.25 x 2.00 | 1.20 x 1.20 x 1.20 |
| Field of view (mm) | 308 x 299 | 252 x 316 |
| Slice | 160 | 260 |
| TR (ms) | 2200 | 2200 |
| TEequiv (ms) | 64 | 100 |
| TSE factor | 39 | 56 |
| Refocusing pulses | NA | Constant |
| Min angle(°) | NA | 50 |
| Max angle(°) | NA | 50 |
| Fat suppression. | STIR | SPAIR |
| Inversion recovery delay (ms) | 270 | 210 |
| Pulse type | NA | NA |
| MSDE |  |  |
| Refocusing type | Adiabatic | Adiabatic |
| VENC (cm/s) | 1.5 | 0.5 |
| TEprep pulse | 35 | 50 |
| Time | 7 min 40 s | 6 min 23 s |

*Abbreviations.* MSDE, motion-sensitive driven equilibrium; OIT, offset independent trapezoid; SPAIR, Spectral Attenuated Inversion Recovery; STIR, short TI inversion recovery; TEequiv, echo time equivalent; TEprep, echo time preparation; TR, repetition time; TSE, turbo spin-echo; VENC, velocity encoding; NA, not applicable.

**Table A2. Studies reporting the delineation of peripheral nerves with MR/CT registration technique**

| Study | Patients | Malignancy | NOI | MR Series | MR Thickness | Registration |
| --- | --- | --- | --- | --- | --- | --- |
| Truong, et al.,  2010 [28] | /^*^ | Not mentioned | BP | Coronal T1 | /^#^ | Rigid |
| Biau, et al.,  2019 [29] | /^*^ | H&N cancers | CN V1-3 | Post Gado T1 | /^#^ | Rigid |
| Li, et al.,  2019 [30] | 15 | NPC | BP | T1 or T2 | ≥3.0mm | Rigid |
| Hwang, et al., 2021 [31] | 35 | PC | Periprostatic NVBs | T2 | 3.0-4.5mm | Rigid |
| Present study | 18 | PC | LSP and  its components | NerveVIEW | 2.0mm | Deformable |

*Abbreviations*: NOI, nerve of interest; BP, brachial plexus; H&N, head and neck; CN V1-3, trigeminal nerve and its branches; NPC, nasopharyngeal cancer; NVB, neurovascular bundle; LSP, lumbosacral plexus. ^*^ The number of patients enrolled was not mentioned in the article. ^#^ The slice thickness of MR was not mentioned in the article.
